# Early stage Acute B lymphocytic leukemia presenting with symptoms of ankylosing spondylitis (AS): A case report

**DOI:** 10.1097/MD.0000000000019806

**Published:** 2020-04-10

**Authors:** Wei Liu, Guangfeng Chen, Bing Xu, Suping Sun, Jingzhen Tian, Yingying Zhang

**Affiliations:** a College of Traditional Chinese Medicine, Shandong University of Traditional Chinese Medicine; b Department of Rheumatology, The Affiliated Hospital of Shandong University of Traditional Chinese Medicine, Jinan; c Qingdao Academy of Traditional Chinese Medicine, Shandong University of Traditional Chinese Medicine, Qingdao, China.

**Keywords:** acute lymphoblastic leukemia, ankylosing spondylitis, case report, fever, HLA_B27, joint pain, lymphocytes, lymphocytes percentage, misdiagnose, rheumatic disease, young patients

## Abstract

**Rationale:**

Acute lymphoblastic leukemia (ALL) has acute and severe onset characterized by fever, moderate to severe anemia, bone and joint pain, and sternal tenderness. It is easy to be misdiagnosed as rheumatic disease when joint pain is the first symptom.

**Patient concerns:**

A male Han, 18 years of age was admitted on July 15th, 2016 for multi-joint swelling and pain with intermittent fever for half a year which had aggravated in the last 10 days.

**Diagnosis:**

Based on symptoms, imaging, family history, and blood tests, he was first diagnosed with ankylosing spondylitis, but he was refractory to treatment. Bone marrow biopsy then revealed acute B-lymphoblastic leukemia (possibility Pro-B-ALL).

**Interventions:**

The patient was transferred to the hematology department on July 23rd, 2016 for chemotherapy.

**Outcomes:**

No joint pain occurred during follow-up, which ended on November 4th, 2018.

**Lessons:**

ALL may present with symptoms suggestive of rheumatic diseases like ankylosing spondylitis. Physicians should be aware of this possibility, especially in young patients.

## 1 Introduction

Acute lymphoblastic leukemia (ALL) is a cancer of blood cells in which neoplastic cells morphologically and immunophenotypically resemble B-lineage and T-lineage precursor cells (lymphoblasts).^[1]^ The peak incidence is at 2 to 5 years of age. Total incidence is 1 per 2000 children aged 0 to 15 years and 17 per million in teenagers 15 to 19 years of age.^[2]^

It has acute and severe onset characterized by fever, moderate to severe anemia, bone and joint pain, and sternal tenderness.^1^,^3^ It is easy to be misdiagnosed as rheumatic disease when joint pain is the first symptom.^4^,^5^,^6^,^7^,^8^,^9^,^10^

We present a patient with acute B-lymphocytic leukemia who was misdiagnosed as ankylosing spondylitis (AS) at the rheumatology department of our hospital.

## 2 Case report

A male Han, 18 years of age was admitted on July 15th, 2016 to the rheumatology department for multi-joint swelling and pain with intermittent fever for half a year, aggravating for 10 days. Six months before, the patient had alternating pain of bilateral knee and shoulder joints, with intermittent seizures, and fever with pain, but without clear diagnosis or treatment. Two months ago, he sought medical advice from the rheumatology department due to swelling and pain of both knee and ankle joints with pain of both hip joints. Examinations revealed HLA-B27(+), erythrocyte sedimentation rate 67 mm/h (0–20 mm/h), and C-reactive protein 48.8 mg/L (0–8 mg/L). Sacroiliac joint magnetic resonance imaging (MRI) showed abnormal signals of the right femoral head, bilateral tibia and femur, and spine. He received a diagnosis of AS and received diclofenac sodium, Lutei Dihuang pills, acupuncture and other symptomatic treatments. Later, he was given intra-articular injection of drugs at another hospital (specifically unknown) to the hip and knee joints. The pain was alleviated and resumed. Ten days prior to hospitalization, the patient was treated for aggravation of left hip joint pain, fever, and difficulty walking.

The admission symptoms were severe pain in left hip joint, aggravation at night and difficulty in walking; pain in both ankles and left knee joints; high skin temperature; irregular fever, with maximum body temperature of 38.5°C; and obvious morning stiffness. There was a family history of AS. Physical examinations revealed positive left “4” test, negative right “4” test, positive left hip test, and bilateral ankle and left knee joint swelling and tenderness.

For blood routine, white blood cells were 6.69 × 10^9^/L (reference: 3.50–9.50 × 10^9^/L), neutrophils were 1.58 × 10^9^/L (reference: 1.80–6.30 × 10^9^/L), lymphocytes were 4.2 × 10^9^/L (reference: 1.10–3.20 × 10^9^/L), lymphocytes percentage was 62.6% (reference: 20.0%–50.0%), and hemoglobin was 111 g/L (reference: 115–150 g/L). Both rheumatoid factor and anti-cyclic citrullinated peptide antibody were negative. C-reactive protein was 129 mg/L (reference: 0.00–8.00 mg/L) and anti-streptolysin O was 61.7 IU/mL (reference: 0–408 IU/mL). Antinuclear antibody quantification was negative. Procalcitonin was 0.63 ng/ml (reference: 0–0.5 ng/ml), erythrocyte sedimentation rate was 82 mm/L (reference: 0–20 mm/L), uric acid was 596 μmol/L (reference: 155–357 μmol/L), ferritin was 484.8 ng/mL (reference: 13–150 ng/mL), and lactate dehydrogenase was 252 U/L (reference: 109–249 U/L). Antinuclear antibody spectrum, brucella agglutination test, Epstein-Barr virus, and HLA-B27 were all negative.

**Figure 1:**
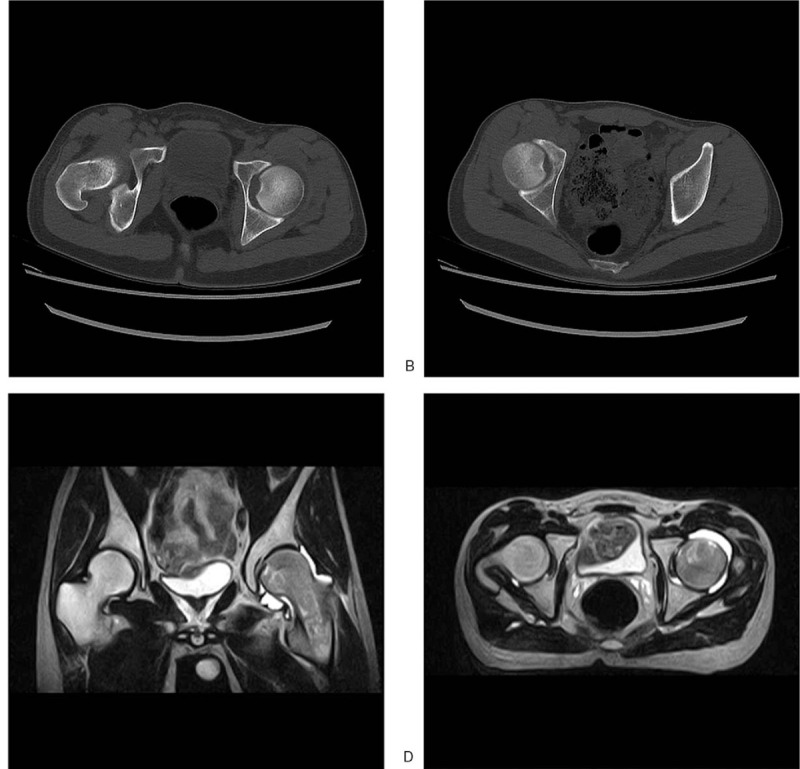
Computed tomography (CT) of the hip joint on July 18, 2016 and magnetic resonance imaging (MRI) of the hip joint on July 19, 2016. *A and B*, CT shows the bilateral hip joint space is narrowed, the articular surface is still intact, the bone trabecular patchy density under the articular surface of the right femoral head is increased uniformly, effusion can be seen in the left hip joint capsule and the left femoral head has no obvious abnormalities. Right femoral head ischemic necrosis and left hip joint effusion were considered. *C and D*, MRI shows the position of the hip joint is normal, the joint space is narrowed, the signal of articular cartilage is not uniform, and the articular surface is intact. The signal of the left femoral head, femoral neck and upper femur is not uniform. The signal of the left pubic muscle and external obturator muscle, local iliopsoas muscle and lateral femoral muscle is slightly increased. Under the cartilage of the right femoral head, patchy long T1 and long T2 and high lipid pressure signal were observed. No abnormal signals were found in the bilateral acetabulum, right femoral neck and upper femur. The T1 signal of bone seen in the scanning range decreased uniformly and the T2 lipid pressure showed uniform high signal. Fluid signals were seen in the pelvis.

Intravenous drips of 30 mL reduning injection (Kangyuan Pharmaceutical Industry, Jiangsu, China) and 20 mL safflower injection (Huawei Pharmaceutical Industry, Shanxi, China) were given, and 75 mg diclofenac sodium capsule were orally taken twice a day (German Taimuler Company, Germany). On the 3rd day of admission, the patient had recurrent severe pain. White blood cells were 2.62 × 10^9^/L and lymphocyte percentage was 61.8%, while anti-nuclear antibody quantification was positive and nuclear granular type was 1:100. Computed tomography (CT) of hip joint showed ischemic necrosis of right femoral head and effusion of left hip joint (Fig. 1). Abdominal color Doppler ultrasonography showed mild splenomegaly and bilateral renal parenchymal enhancement. MRI of hip joint showed left femoral head, bone marrow edema and joint effusion at the upper end of the femoral neck and head, with swelling of surrounding soft tissue and that bone seen was diffused with low signal of T1W and high signal of lipid pressure and pelvic effusion (Fig. 1).

Bone marrow analysis showed: good staining; active bone marrow hyperplasia; granulocyte and erythrocyte hyperplasia decreased significantly; mature red blood cells varied in size; mature lymphocyte accounted for 58%;. promyelocytes accounted for 31.5%, which were round or quasi-circular, with nucleus round or irregular, easy to see the notch, thick chromatin, visible nucleoli, less cytoplasm, sky blue Peroxidase Stain (POX) staining negative; and 1 megakaryocyte was seen in whole blood and platelets scattered in rare. According to immunophenotyping report of hematological tumors, acute B-lymphoblastic leukemia (possibility Pro-B-ALL) was considered. Immunophenotyping showed that lymphocyte was 14.3%, myeloid cells was 21.7%, abnormal cells was 56.2%, and nucleated red blood cells was 7.8%; with CD34+ cells accounting for 56% of nuclear cells, considering acute B lymphocytic leukemia.

The patient was transferred to the hematology department on July 23, 2016. The course of treatment is shown in Table 1. No joint pain occurred during follow-up, which ended on November 4, 2018.

**Table 1:**
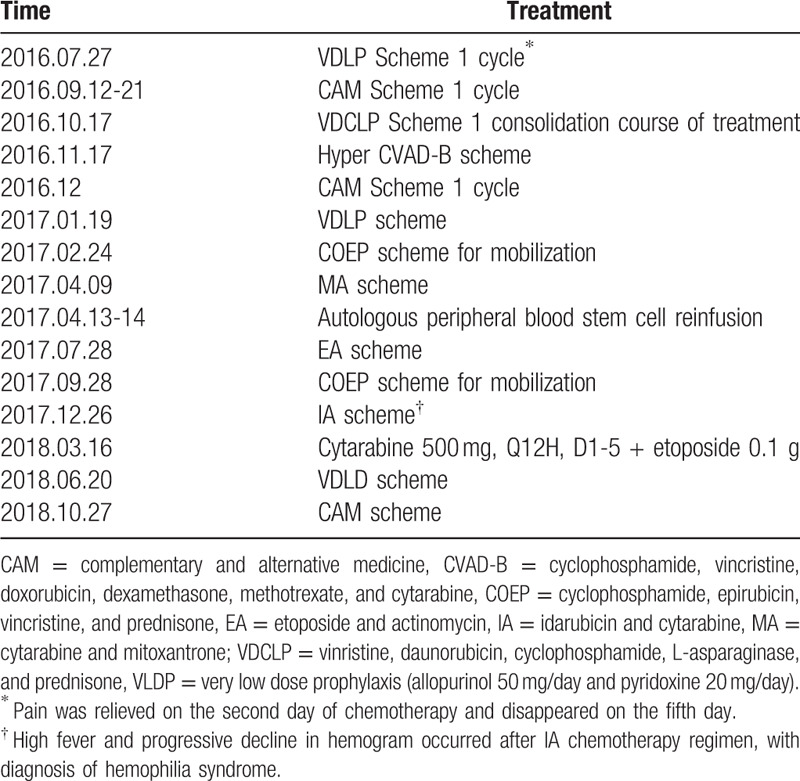
Therapeutic regimen.

## 3 Discussion

Before the patient came to our hospital for treatment, he was diagnosed as AS, which could be supported by:

1. prominent joint manifestations (bilateral hip, knee, and shoulder joint pain, and swelling and pain of both ankle joints);
2. family history of AS; and
3. HLA-B27-positive.

In China, 24% to 75% of AS patients have peripheral joint lesions at the beginning or the course of the disease, with knee, hip, ankle and shoulder joint lesions mostly. The hip joint is the most susceptible peripheral joint in AS.^[11]^ Due to the growth and development of adolescents, the main early symptoms of adolescent AS may not be low back pain and morning stiffness, but symptoms of arthritis or synovitis.^[12]^

On the other hand, some points did not support the diagnosis of AS and prompted further testing:

1) acute onset, rapid development of joint symptoms, affecting functional activities in a short period of time, accompanied by high fever;
2) non-steroidal anti-inflammatory drugs (NSAIDs) alleviated joint pain, but without significant improvement on fever;
3) atypical manifestations of AS on MRI of sacroiliac joint and bilateral hip joints; and
4) clear decrease of white blood cells in the absence of immunosuppressive agents. In the early stage of AS, the anterior and inferior synovium of sacroiliac joint is often involved, and the iliac side is more severer.^11^,^12^

Edema around the joints, abnormal synovial signal, and bone marrow edema are the earliest changes in AS.^[13]^

Hence, the causes of misdiagnosis could be multiple. First, some doctors may have an insufficient understanding of HLA-B27. In China, the positive rate of B27 is as high as 91% in patients with AS, but it is improper to consider HLA-B27 as the gold standard for the disease, other clinical indicators and clinical symptoms should also be considered for comprehensive diagnosis.^11^,^12^ Second, after intra-articular drug injection, the pain was relieved quickly after administration, but glucocorticoid receptors on the ALL lymphocyte surface makes them sensitive to glucocorticoids.^[14]^ Therefore, the disease was relieved, joint swelling and pain disappeared, concealing the condition and making diagnosis difficult. Third, AS is an autoimmune disease and the diagnosis is complex. The first doctor needs to make a full differential diagnosis and exclude other diseases before making a diagnosis. Finally, Rheumatoid manifestations can be found in both paraneoplastic syndrome and leukemia, which is apt to misdiagnose. However, in this case, the rheumatoid manifestations associated with paraneoplastic disease are not related to the direct infiltration of tumor cells, so paraneoplastic syndrome is excluded.^15^,^16^ On the other hand, according to imaging diagnosis, the patient's hip joint disease is caused by tumor cell infiltration of leukemia, and its clinical manifestations do not conform to the pathological characteristics caused by AS,^[17]^ so the final diagnosis is leukemia.

In conclusion, when reviewing the whole process of diagnosis and treatment, we believe that bone marrow examination should be done early for young male patients with rapid onset of joint pain accompanied by fever, to avoid clinical misdiagnosis, even if peripheral blood cells are normal.

## Acknowledgments

The authors acknowledge the help of the Collaborative Innovation Center of the Chinese Medicine Anti-virus in Shandong University of Traditional Chinese Medicine.

## Author contributions

**Conceptualization:** Wei Liu.

**Data curation:** Guangfeng Chen.

**Formal analysis:** Wei Liu, Bing Xu, Yingying Zhang.

**Methodology:** Guangfeng Chen, Suping Sun.

**Project administration:** Bing Xu, Jingzhen Tian.

**Resources:** Guangfeng Chen.

**Supervision:** Jingzhen Tian.

**Validation:** Suping Sun.

**Writing – original draft:** Wei Liu, Guangfeng Chen.

**Writing – review & editing:** Bing Xu, Suping Sun, Yingying Zhang.
